# Severely Burdened Individuals Do Not Need to Be Excluded From Internet-Based and Mobile-Based Stress Management: Effect Modifiers of Treatment Outcomes From Three Randomized Controlled Trials

**DOI:** 10.2196/jmir.9387

**Published:** 2018-06-19

**Authors:** Kiona Krueger Weisel, Dirk Lehr, Elena Heber, Anna-Carlotta Zarski, Matthias Berking, Heleen Riper, David Daniel Ebert

**Affiliations:** ^1^ Department of Clinical Psychology and Psychotherapy Institute of Psychology Friedrich-Alexander-University Erlangen-Nuernberg Erlangen Germany; ^2^ Division of Online Health Training Innovation Incubator Leuphana University Lueneburg Lueneburg Germany; ^3^ Department of Health Psychology and Applied Biological Psychology Institute for Psychology Leuphana University Lueneburg Lueneburg Germany; ^4^ GET.ON Institute for Online Health Trainings Hamburg Germany; ^5^ Department of Clinical, Neuro and Developmental Psychology Faculty of Behaviour and Movement Sciences VU University Amsterdam Amsterdam Netherlands

**Keywords:** anxiety, depression, internet, effect modifier, randomized controlled trials, occupational stress

## Abstract

**Background:**

Although internet-based and mobile-based stress management interventions (iSMIs) may be a promising strategy to reach employees suffering from high chronic stress, it remains unknown whether participants with high symptom severity of depression or anxiety also benefit from iSMIs or should be excluded.

**Objective:**

This study aimed to evaluate the efficacy of iSMIs in subgroups with high symptom severity and to test whether baseline symptom severity moderates treatment outcome.

**Methods:**

Data from three randomized controlled trials (N=791) were pooled to identify effect modifiers and to evaluate efficacy in subgroups with different levels of initial symptom severity. The outcomes perceived stress (Perceived Stress Scale, PSS), depression severity (Center for Epidemiological Depression Scale, CES-D), and anxiety (Hospital Anxiety and Depression Scale, HADS) symptom severity were assessed at baseline, 7-week postassessment, and 6-month follow-up. Potential moderators were tested in predicting differences in the change of outcome in multiple moderation analyses. Simple slope analyses evaluated efficacy of the iSMI comparing the intervention group with the waitlist control group in subgroups with low, moderate, and severe initial symptomology based on means and SDs of the study population. In addition, subgroups with clinical values of depression (CES-D≥16) and anxiety (HADS≥8) at baseline were explored, and response rates (RRs; 50% symptom reduction) and symptom-free (SF) status (CES-D<16, HADS<8) were reported.

**Results:**

Individuals with high stress (PSS≥30), depression (CES-D≥33), anxiety (HADS≥15), and emotional exhaustion (MBI≥5.6) benefited significantly from the intervention with great reductions of stress (*d*_post_=0.86-1.16, *d*_FU_=0.93-1.35), depression (*d*_post_=0.69-1.08, *d*_FU_=0.91-1.19), and anxiety (*d*_post_=0.79-1.19, *d*_FU_=1.06-1.21), and effects were sustained at 6-month follow-up. Symptom severity moderated treatment outcomes, as individuals with higher symptom severity at baseline benefited significantly more from the intervention than individuals with lower symptom severity. Furthermore, 82.9% (656/791) of individuals had clinical depression values at baseline, of which significantly more individuals in the intervention group reached at least 50% symptom reduction or fell under clinical cut-off (RR: 29.2%, 93/318; SF: 39.6%, 126/318) compared with the waitlist control group (RR: 8.0%, 27/338; SF: 18.6%, 63/338) at postassessment. Significantly more individuals with clinical anxiety values at baseline (HADS≥8, 85.3%, 675/791) in the intervention group achieved at least 50% symptom reduction or fell under clinical cut-off (RR: 27.7%, 94/339; SF: 39.8%, 135/339) compared with the WLC (RR: 4.8%, 16/336; SF: 15.5%, 52/336).

**Conclusions:**

Highly burdened individuals benefit greatly from iSMIs and therefore should not be excluded from participation. Stress management may be a valid entry point to reach highly burdened individuals who otherwise may not seek treatment.

**Trial Registration:**

1) German Clinical Trials Register DRKS00005112; https://www.drks.de/DRKS00005112 (Archived by WebCite at http://www.webcitation.org/6zmIZwvdA); 2) German Clinical Trials Register DRKS00005384; https://www.drks.de/ DRKS00005384 (Archived by WebCite at http://www.webcitation.org/6zmIerdtr); and 3) German Clinical Trials Register DRKS00004749; https://www.drks.de/DRKS00004749 (Archived by WebCite at http://www.webcitation.org/6zmIjDQPx).

## Introduction

### Background

High chronic stress is linked to adverse psychological health outcomes. Left untreated, individuals suffering from high occupational stress can develop common mental disorders such as depression or anxiety [1]. Stress, depression, and anxiety are associated with productivity loss and absenteeism, and can negatively affect workplace safety [2,3].

Meta-analytic evidence on occupational interventions of the last two decades, aiming to improve mental health, has found varying evidence of benefit ranging from nonsignificant to moderate effect sizes depending on, for example, type of intervention, intervention content, and outcome categories [4,5]. In a systematic review, Martin et al [6] found overall small positive effects in pooled data of 17 studies, investigating whether different types of health promotion interventions in the workplace reduce depression (standardized mean difference, SMD=0.28, 95% CI 0.12-0.44) and anxiety (SMD=0.29, 95% CI 0.06-0.51). A systematic review and meta-analysis from 2014 identified nine workplace-based randomized controlled trials (RCTs) aimed at reducing the level of depression symptoms. Pooled effect size estimates showed the interventions to be superior to the control groups by a small positive effect (SMD=0.16, 95% CI 0.07-0.24) [7,8]. Richardson et al found a moderate effect size across studies in a meta-analysis of stress management interventions (SMIs) in occupational settings (*d*=0.53, 95% CI 0.36-0.69), significant moderate intergroup effects for anxiety (*d*=0.68), and a small to moderate effect for mental health (*d*=0.44) [9]. Although the range of effect size varies, occupational interventions and SMIs seem to bear the potential to improve psychological health outcomes. However, the previous studies did not examine whether there were differences in efficacy between subgroups with varying initial symptom severity. It therefore remains an open research question whether individuals with high clinical symptomology, that is, depression and anxiety, also benefit from SMIs.

One specific form of SMIs, which has proven to be effective in certain contexts, is internet-based and mobile-based stress management interventions (iSMIs) [5,10]. Advantages of iSMIs include the following: (1) individuals can avoid stigmatization by participating anonymously, (2) such trainings are flexible and adaptable to any work and life situation, (3) material can be revised as often as desired, (4) access to treatment and treatment uptake is facilitated by not having waiting times nor a limitation of resource distribution, and (5) fostering self-efficacy of participants.

Occupational iSMIs could reach individuals who would likely not seek psychological treatment [11,12]. This includes severely burdened individuals who, thus, have developed clinical profiles with high symptoms of depression and anxiety. Most individuals with depression and anxiety do not seek treatment, for example, because of fear of stigma [13]. SMIs may bear the potential to attract individuals who would not make use of mental health interventions explicitly labeled for targeting mental health, that is, depression or anxiety. However, it remains unknown whether individuals with high symptom severity also benefit from low threshold iSMIs, as its exploration, to date, is lacking.

It seems plausible that individuals with severe symptomology, for example, those who experience clinical levels of depression or anxiety, are too burdened to substantially improve their mental health through occupational SMIs not specifically designed to treat depression or anxiety. It may be that methods and techniques delivered in SMIs are not sufficient, as highly burdened participants may need more therapeutic support than generally provided in (i)SMIs. In addition, with regard to iSMIs, some individuals with high symptom severity may be overwhelmed and unable to apply psychological self-help strategies effectively into their daily lives.

Allowing severely affected individuals to participate in (i)SMIs, who are unlikely to benefit, could be problematic. Participation may result in aggravation, hopelessness, and deterioration of symptoms, and may delay, or in the worst case, inhibit affected individuals from seeking appropriate treatment in time, hence contributing to a chronification of symptoms [14]. In addition, this may result in unnecessary intervention delivery costs. Thus, it is crucial to investigate differential effects of SMIs, and to test whether such approaches are also effective in severely affected populations, or whether these individuals should rather be excluded from SMIs and referred to clinical treatment for psychological disorders.

Through moderation analysis, differential treatment effects can be investigated. However, primary studies are generally solely powered to detect overall treatment effects, and thus underpowered to adequately perform reliable subgroup and moderator analyses [15]. To overcome this issue, data can be pooled from single studies by adding individual participant data in one dataset for common analyses [16].

### Objective

This study aims to investigate the effects of an iSMI in subgroups of individuals who experience severe levels of stress, depression, anxiety, emotional exhaustion, or insomnia at baseline, and to test whether these baseline indicators of clinical impairment moderate the intervention efficacy in the reduction of stress, depression, and anxiety. If proven effective for severely burdened individuals, iSMIs could be a crucial component in the amelioration of mental health in occupational health settings, irrespective of the severity level of clinical impairment populations experience.

## Methods

### GET.ON Stress Intervention

Secondary analyses were conducted based on pooled individual participant data from three RCTs evaluating the same iSMI (GET.ON Stress). The three studies comparing an iSMI to a waitlist control group (WLC) were identical in design, differing only in respect to guidance intensity [17]. The iSMI is based on the Lazarus and Folkman’s transactional model of stress [18], and core components focus on problem solving [19,20] and emotion regulation [21,22]. The studies were approved by the ethics committee of the Philipps University Marburg (2013-20K, AZ 2013-35K, AZ 2012-43K) and registered in the German Clinical Trial Register under DRKS00005112, DRKS00005384, and DRKS00004749. Further details on the intervention can be found in a published study protocol [23].

### Sample

Participants of the three studies were recruited via the occupational health program of a large health insurance company in Germany (company website, newspaper articles, and advertisements in the membership magazine), a study website, mass media (newspapers and television), and announcements by the Ministry of Education. Recruitment was open to the general working population and not restricted to members of a certain health insurance company. In total, 791 participants were included. Inclusion and exclusion criteria are provided in Textboxes 1 and 2, respectively.

**Figure 1:**
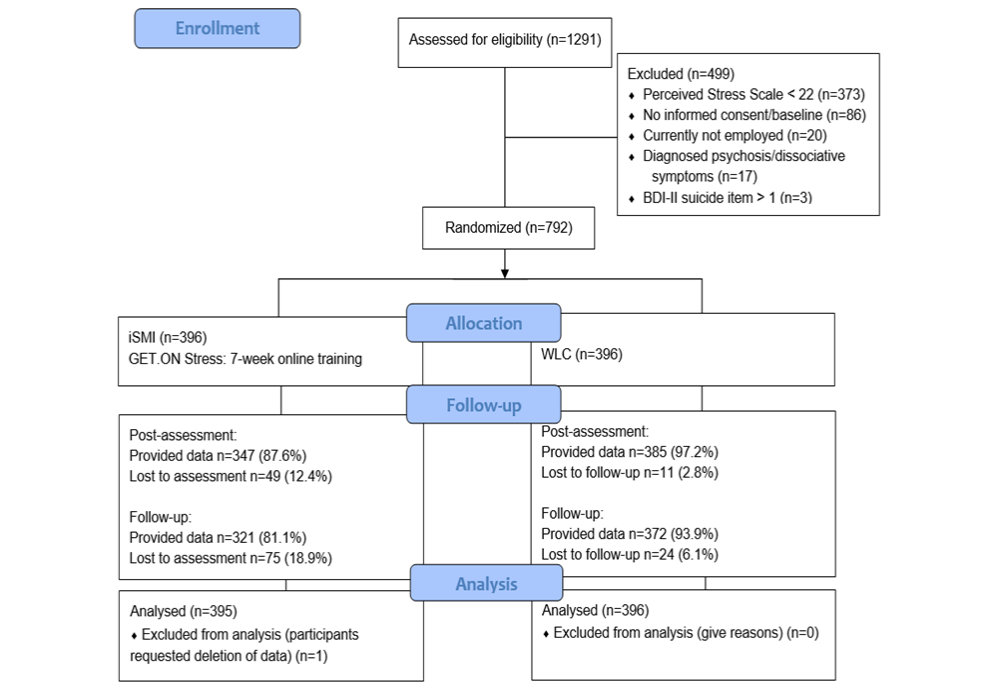
Flow diagram of participants. BDI: Beck Depression Inventory; iSMI: internet-based and mobile-based stress management intervention; WLC: waitlist control group.

A standard procedure was followed when individuals showed a notable suicidal risk. They were advised to seek help from their general practitioner, local psychiatric emergency room, or to contact the official emergency number. Telephone numbers and information on relevant institutions were provided via email. The cut-off on the Perceived Stress Scale (PSS) was chosen to include participants with heightened stress based on 1 SD (6.2) above the mean (15.3) found in a large working population [26]. There was no participation cut-off for individuals with severe impairment because of critically high symptom severity of depression or anxiety. Figure 1 shows the flow of participants.

### Measures

The outcomes stress, depressive symptom severity, and anxiety symptom severity were collected by self-report at baseline (T1), at postassessment 7 weeks after randomization (T2), and at 6-month follow-up (T3). Furthermore, potential moderators, emotional exhaustion and insomnia severity, were collected at baseline.

### Primary Outcome

The primary outcome of the primary studies was perceived stress measured by the German version of the PSS (10 items; score range: 0-4; total score range: 0-40; alpha_T1_=.75) [22,24]. Higher scores on the PSS indicate more severe perceived stress.

**Inclusion criteria for the study.**

- Currently employed
- Aged 18 years or older
- Scores of 22 or above on Perceived Stress Scale [22,24]
- Internet access and a valid email address
- Sufficient reading and writing skills in German
- Willing to give informed consent

**Exclusion criteria for the study.**

- If the participants self-reported having been diagnosed with psychosis or dissociative symptoms
- If the participants showed a notable suicidal risk indicated by a score greater than 1 on the Beck suicide item [25]

### Secondary Outcomes

Secondary outcomes included depressive symptom severity measured by the German version of the frequently used Center for Epidemiological Depression Scale (CES-D; 20 items; score range: 0-3; total score range: 0-60; alpha_T1_=.86) [27]. Scores of CES-D≥16 indicate clinically relevant levels of depression severity [28]. Anxiety symptom severity was measured by the anxiety subscale of the Hospital Anxiety and Depression Scale (HADS; 7 items; score range: 0-3; total score range: 0-21; alpha_T1_=.74) [29]. A cut-off of ≥8 indicates clinically relevant levels of anxiety [30].

Additional mental health assessments included emotional exhaustion, the basic stress dimension of burnout, measured by the German Version Maslach Burnout Inventory (MBI; 5 items; score range: 1-6; total score range 1-6; alpha_T1_=.78) [31,32], and insomnia severity, assessed by the Insomnia Severity Index (ISI; 7 items; score range: 0-4; total score range 0-28; alpha_T1_=.82) [33]. Higher scores on all secondary outcome scales indicate more severe or higher symptoms than lower scores.

### Statistical Analysis

#### Missing Data

Treatment outcomes included perceived stress, depressive symptom severity, and anxiety symptoms. The dropout rate ranged from 7.5% (59/791) in the primary outcome stress at postassessment to 13.8% (109/791) at 6-month follow-up in the outcome insomnia severity. Overall, these are low rates of intervention dropout. Comparing the dropout rates between the iSMI and the WLC, it becomes evident that the outcome dropout rates are higher in the intervention condition (12.4%-21%) compared with the WLC (2.5%-6.6%).

Missing data of all outcomes were handled with multiple imputations (MIs) in accordance with the intention-to-treat principle [34,35]. Prerequisites of MI include that the data are missing at random and that the data are normally distributed. Using Little’s missing completely at random (MCAR) test, it was determined that the missing data were MCAR. The test was not significant (χ^2^_50_=40.2, *P*=.84). However, the outcome data were not normally distributed, which can potentially lead to implausible low or negative values after using MI. To verify post hoc that this did not affect the imputed data, it was tested whether outliers in the new dataset corresponded with missing data in the original dataset, which was not the case.

Missing data were handled by using the MI procedure in SPSS. Estimations of missing data were based on all available data for all outcome measures at all assessment points (T1, T2, and T3), as well as age, gender, and intervention condition (iSMI and WLC). In addition, 100 imputed datasets were created and aggregated into 1 dataset for the final analysis. Significance level was set to <.05.

#### Multiple Moderation Analyses

To identify subgroups with different levels of initial symptom severity and to assess the efficacy of the intervention for populations with severe impairment on changes in outcomes (stress, depressive, and anxiety symptoms), multiple moderation analyses (MMAs) were performed with the SPSS macro PROCESS [36]. MMAs are separate multiple regression models each containing 3 elements that are tested in 3 steps predicting the outcome: (1) main effect of the baseline variable, (2) main effect of the treatment condition (iSMI and WLC), and (3) interaction effect (baseline×treatment condition). If the interaction effect is significant in predicting the outcome, it is considered to be an effect modifier moderating the outcome. Effect modifiers of treatment outcome indicate that populations benefit significantly different from each other depending on initial symptom severity. Moderators of the difference in change of the outcomes will be reported for postassessment and 6-month follow-up.

To explore the direction of an interaction effect, improve interpretability, and provide specific estimations for the investigated subgroups of interest, follow-up simple slope analyses were performed. Baseline data on perceived stress, depression, anxiety, emotional exhaustion, and insomnia severity were split into 3 subgroups based on distributional values, mean (M), and SD of the study population (mean-1SD; mean; mean+1SD), and outcomes in each subgroup were explored. To compare efficacy of the iSMI and WLC in the respected subgroups at postassessment and 6-month follow-up, Cohen *d*, 95% CI, and the number needed to treat (NNT) were calculated. Baseline differences were controlled for by using the difference in changes of outcome from baseline to postassessment (T1-T2) and from baseline to 6-month follow-up (T1-T3) as dependent variable. The study variable was not included in the final analysis, as it was not associated with the primary outcome stress at 6-month follow-up. Cohen *d* can be interpreted based on 3 approximations: small effects (*d*=0.2), medium effects (*d*=0.5), and large effects (*d*=0.8) [37]. All continuous variables were standardized for interpretability.

#### Response Rates and Symptom-Free Status in Clinical Subgroups

In addition, effects in subgroups with clinical values of depression (CES-D≥16) and anxiety (HADS≥8) at baseline were explored. Results were reported as mean between-group differences, Cohen *d*, and NNT while controlling for baseline severity by using change scores from baseline to postassessment. Improvements at an individual level were investigated by assessing the number of participants who achieved a response rate (RR) through 50% symptom reduction and symptom-free (SF) status (CES-D<16, HADS<8). Differences between the intervention condition and control group were tested in a chi-square (χ^2^) test.

## Results

### Descriptive Statistics

Participants were aged 42.6 years (SD 9.7) on average at baseline, and the majority were female (76.7%, 607/791). Overall, the sample was highly educated with a minimum of 12 years of schooling and a degree (71.9%, 569/791) or mid-level education with 10 years of schooling and a degree (25.4%, 201/791), with only 2.5% (21/791) of persons having low education levels with no school degree or 9 years of schooling. Participants had an average value of 25.6 (SD 4.3) on the PSS. The mean of depressive symptomology (CES-D) was 23.9 (SD 8.6). Anxiety symptoms (HADS) were at an average of 11.1 (SD 3.4). The average of emotional exhaustion (MBI) was 4.7 (SD 0.7). Insomnia severity (ISI) was at a mean of 13.7 (SD 6.1). Most participants had clinically relevant levels of depression (82.9%, 656/791) and anxiety (85.3%, 675/791) at study uptake. Further details on baseline characteristics can be found in Table 1.

The classification in subgroups with low, moderate, and severe initial symptom severity is based on means and SDs of the study population. Means and SDs for all investigated subgroups (mean-1SD; mean; mean+1SD) with regard to all potential predictors (perceived stress [PSS], depressive symptoms [CES-D], anxiety [HADS], emotional exhaustion [MBI], and insomnia severity [ISI]), and outcomes (PSS, CES-D, and HADS) can be found in Table 2. All subgroups showed a reduction of symptom severity in favor of the iSMI in comparison with the WLC, and effects were sustained at 6-month follow-up.

### Primary Outcome

**Figure 2:**
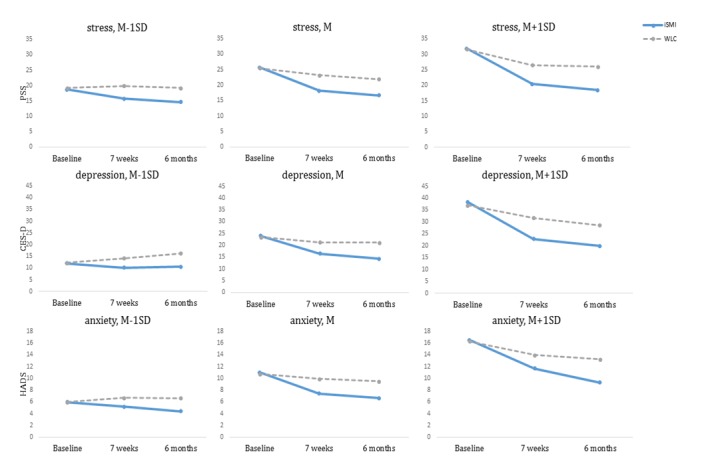
Simple slope: illustrative course of estimated course of symptom change in waitlist control group and internet-based and mobile-based stress management interventions for significant moderator “perceived stress” on stress, “depression severity” on depression, and “anxiety severity” on anxiety between baseline, postassessment, and 6-month follow-up. M: mean; PSS: Perceived Stress Scale; CES-D: Center for Epidemiological Depression Scale; HADS: Hospital Anxiety and Depression Scale; iSMI: internet-based and mobile-based stress management interventions; WLC: waitlist control group.

Effect sizes for differences in change of perceived stress at postassessment were medium to large. The smallest effect was observed for individuals with low emotional exhaustion at baseline (*d*=0.57; 95% CI 0.22-0.91; NNT=3.18), and greatest effects were observed for those with high emotional exhaustion (*d*=1.16; 95% CI 0.78-1.54; NNT=1.7). Moderators of change in the outcome stress at postassessment were stress (beta=1.04, *P=*.01) and emotional exhaustion (beta=1.12, *P=*.008). Highly stressed individuals and individuals with high emotional exhaustion profited significantly more from the iSMI than individuals with low symptom severity (low: PSS_T1-T2_*d*=0.65, MBI_T1-T2_*d*=0.57; high: PSS_T1-T2_*d*=0.98, MBI_T1-T2_*d*=1.16). Figure 2 shows the estimated course of symptom change in the iSMI compared with the WLC at postassessment and 6-month follow-up.

Looking at the effects at 6-month follow-up, moderate to large effects were found in change of stress, the smallest effect for stress (*d*=0.66; 95% CI 0.3-1.02; NNT=2.78) and the largest effect for emotional exhaustion (*d*=1.35; 95% CI 0.96-1.75; NNT=1.52). Stress (beta=0.96, *P*=.049) was the only moderator for change in stress at 6-month follow-up. Highly stressed individuals showed greater reduction in stress through the intervention than low-stressed individuals (low: PSS_T1-T3_*d*=0.66, high: PSS_T1-T3_*d*=1.27).

**Table 1:** Baseline characteristics of the study population.

Characteristics		All (N=791)	iSMI^a^ (n=395)	WLC^b^ (n=396)
Age in years, mean (SD)		42.6 (9.7)	42.1 (9.9)	43.1 (9.5)
**Gender, n (%)^c^**				
	Female	607 (76.7)	307 (77.5)	300 (75.9)
	Male	181 (22.9)	87 (22)	94 (23.8)
	Other	2 (0.3)	1 (0.3)	1 (0.3)
**Ethnicity, n (%)**				
	Caucasian or white	657 (83.1)	326 (82.3)	331 (83.8)
	Other or no information	134 (16.9)	70 (17.7)	64 (16.2)
**Marital status, n (%)**				
	Unmarried	230 (29.1)	122 (30.8)	108 (27.3)
	Married	376 (47.5)	191 (48.2)	185 (46.8)
	Cohabited	95 (12)	51 (12.9)	44 (11.1)
	Separated or divorced	82 (10.4)	30 (7.6)	52 (13.2)
	Widowed	8 (1)	2 (0.5)	6 (1.5)
**Education, n (%)**				
	Low	21 (2.7)	11 (2.8)	10 (2.5)
	Middle	201 (25.4)	101 (25.5)	100 (25.3)
	High	569 (71.9)	284 (71.7)	285 (72.2)
**Employment status, n (%)**				
	Permanent	652 (82.4)	322 (81.3)	330 (83.5)
	Temporary	77 (9.7)	44 (11.1)	33 (8.4)
	Self-employed	50 (6.3)	26 (6.6)	24 (6.1)
	Other	12 (1.5)	4 (1)	8 (2)
**Employment situation, n (%)**				
	Full-time	601 (76)	302 (76.3)	299 (75.7)
	Part-time	180 (22.8)	88 (22.2)	92 (23.2)
	Sick leave	10 (1.3)	6 (1.5)	4 (1)
**Occupational sectors, n (%)**				
	Social, education	208 (26.3)	95 (24)	113 (28.6)
	Service provision	156 (19.7)	85 (21.5)	71 (18)
	Finance, administration	129 (16.3)	63 (15.9)	66 (16.7)
	Health	110 (13.9)	63 (15.9)	47 (11.9)
	Iformation technology, computer	48 (6.1)	26 (6.6)	22 (5.6)
	Media	23 (2.9)	8 (2)	15 (3.8)
	Natural sciences	22 (2.8)	8 (2)	14 (3.5)
	Metal, engineering	18 (2.3)	10 (2.5)	8 (2)
	Production, manufacture	15 (1.9)	7 (1.8)	8 (2)
	Construction, architecture	13 (1.6)	3 (0.8)	10 (2.5)
	Social sciences, liberal arts	11 (1.4)	6 (1.5)	5 (1.3)
	Art, culture, and design	10 (1.3)	7 (1.8)	3 (0.8)
	Infrastructure, logistics	10 (1.3)	4 (1)	6 (1.5)
	Technology	7 (0.9)	3 (0.8)	4 (1)
	Agriculture, environment	7 (0.9)	5 (1.3)	2 (0.5)
	Electro	4 (0.5)	3 (0.8)	1 (0.3)
**Gross annual income (in Euro), n (%)**				
	Low (<30,000)	207 (26.2)	116 (29.3)	91 (23)
	Middle (30,000-50,000)	200 (25.3)	99 (25)	101 (25.6)
	High (>50,000)	309 (39.1)	153 (38.6)	156 (39.5)
	Not reported	75 (9.5)	28 (7.1)	47 (11.9)
**Experience with health-related programs, n (%)**				
	Yes	107 (13.5)	49 (12.4)	58 (14.7)
	No	684 (86.5)	347 (87.6)	377 (85.3)
**Experience with face-to-face psychotherapy, n (%)**				
	Yes	311 (39.3)	151 (38.1)	160 (40.5)
	No	480 (60.7)	245 (61.9)	235 (59.5)
PSS^d^, mean (SD)		25.6 (4.3)	25.6 (4.5)	25.5 (4.1)
CES-D^e^, mean (SD)^f^		23.9 (8.6)	23.9 (9)	24 (8.1)
CES-D≥16, n (%)		656 (82.9)	318 (40.2)	338 (42.7)
HADS^g^, mean (SD)		11.1 (3.4)	11.1 (3.4)	11.0 (3.4)
HADS≥8, n (%)		675 (85.3)	339 (42.9)	336 (42.5)
MBI^h^, mean (SD)		4.7 (0.7)	4.7 (0.7)	4.74 (0.7)
ISI^i^, mean (SD)^f^		13.7 (6.1)	13.9 (6.1)	13.6 (6.1)

^a^iSMI: internet-based and mobile-based stress management interventions.

^b^WLC: waitlist control group.

^c^Due to missing data, the incidences refer to a sample of n=790.

^d^PSS: Perceived Stress Scale.

^e^CES-D: Center for Epidemiological Depression Scale.

^f^Baseline data were imputed as intention-to-treat population values were used later in the analysis.

^g^HADS: Hospital Anxiety and Depression Scale.

^h^MBI: Maslach Burnout Inventory.

^i^ISI: Insomnia Severity Index.

Table 3 shows the effects on change in the outcomes stress, depressive symptoms, and anxiety from baseline to postassessment for the 3 subgroups divided by baseline psychopathology (mean-1SD; mean; mean+1SD) of stress, depression, anxiety, emotional exhaustion, and insomnia severity. The table also displays the *P* values of the standardized regression coefficient of the interaction effect between group (iSMI and WLC) and potential moderators. *P* values below .05 indicate that symptom severity of a certain characteristic is a moderator of treatment outcome.

### Secondary Outcomes

Effect sizes for differences in change of depression at postassessment were small to large. The smallest effect was observed in participants with low emotional exhaustion at baseline (*d*=0.37; 95% CI 0.03-0.71; NNT=4.85) and the largest effects for those with high levels of depression severity (*d*=1.08; 95% CI 0.7-1.46; NNT=1.81). Moderators of change in the outcome depression at postassessment were depression (beta=1.89, *P*=.001), stress (beta=2.1, *P*<.001), and emotional exhaustion (beta=2.28, *P*<.001). Participants with initially high depressive symptoms, high perceived stress, and high emotional exhaustion benefitted significantly more from the intervention in reducing depressive symptoms than individuals with lower severity (low: CES-D_T1-T2_*d*=0.67, PSS_T1-T2_*d*=0.49, MBI_T1-T2_*d*=0.37; high: CES-D_T1-T2_*d*=1.08, PSS_T1-T2_*d*=0.92, MBI_T1-T2_*d*=1.01).

**Table 2:** Overview of internet-based and mobile-based stress management interventions (iSMI) and waitlist control group (WLC) symptom severity of subgroups at baseline (T1), postassessment (T2), and 6-month follow-up (T3).

Symptom severity			Mean–1SD		Mean		Mean+1SD	
			n	Mean (SD)	n	Mean (SD)	n	Mean (SD)
**Perceived Stress Scale**								
	**T1**							
		iSMI^a^	68	18.7 (2.6)	255	25.7 (2.2)	73	31.8 (1.9)
		WLC^b^	60	19.2 (2.2)	268	25.4 (2)	67	31.7 (1.9)
	**T2**							
		iSMI	68	15.7 (5.4)	255	18.2 (5.4)	73	20.4 (6.9)
		WLC	60	19.9 (5.2)	268	23.2 (5.3)	67	26.5 (5.8)
	**T3**							
		iSMI	68	14.6 (5.3)	255	16.6 (6)	73	18.4 (6.6)
		WLC	60	19.2 (6.4)	268	21.9 (5.8)	67	26 (6.3)
**Center for Epidemiological Depression Scale**								
	**T1**							
		iSMI	78	11.9 (3.1)	255	23.9 (4.7)	63	38.4 (5)
		WLC	57	12.2 (2.4)	267	23.5 (4.6)	62	37 (3.9)
	**T2**							
		iSMI	78	10.1 (6.2)	255	16.5 (7.4)	63	22.9 (10.9)
		WLC	57	14.1 (5.4)	267	21.2 (7.7)	62	31.7 (8.6)
	**T3**							
		iSMI	78	10.5 (6.8)	255	14.3 (6.9)	63	19.8 (10)
		WLC	57	16.3 (7.3)	267	21.1 (8.7)	62	28.6 (10.7)
**Hospital Anxiety and Depression Scale**								
	**T1**							
		iSMI	57	5.9 (1.2)	279	11 (1.9)	60	16.5 (1.6)
		WLC	59	5.9 (1)	264	10.7 (1.8)	72	16.3 (1.4)
	**T2**							
		iSMI	57	5.2 (2.5)	279	7.4 (3.1)	60	11.7 (3.9)
		WLC	59	6.7 (2.5)	264	9.9 (2.9)	72	14 (3)
	**T3**							
		iSMI	57	4.4 (2.3)	279	6.6 (3.1)	60	9.3 (3.7)
		WLC	59	6.6 (3.2)	264	9.5 (3.4)	72	13.3 (3.6)
**Maslach Burnout Inventory**								
	**T1**							
		iSMI	74	3.6 (0.4)	258	4.8 (0.4)	64	5.8 (0.2)
		WLC	62	3.6 (0.5)	273	4.8 (0.4)	60	5.8 (0.2)
**Insomnia Severity Index**								
	**T1**							
		iSMI	62	4.4 (2.1)	261	13.7 (3.3)	69	22.7 (2.6)
		WLC	71	4.4 (2.1)	249	13.8 (3.4)	74	22.3 (2.1)

^a^iSMI: internet-based and mobile-based stress management interventions.

^b^WLC: waitlist control group.

Effects sizes for differences in change of depression at 6-month follow-up were moderate to large. The smallest effect was found for participants with initially low levels of stress (*d*=0.6; 95% CI 0.24-0.95; NNT=3.05) and the largest effect for participants with high levels of emotional exhaustion (*d*=1.19; 95% CI 0.81-1.58; NNT=1.67). Differences in change of depressive symptoms from baseline to 6-month follow-up were moderated by stress (beta=2.28, *P*<.001), emotional exhaustion (beta=1.91, *P*=.002), and depression (beta=1.41, *P*=.025). Participants with higher levels of stress, emotional exhaustion, and depressive symptoms benefitted more in reducing depressive symptoms (high: CES-D_T1-T3_*d*=1.02, PSS_T1-T3_*d*=1.2, MBI_T1-T3_*d*=1.19) than participants with lower levels (low: CES-D_T1-T3_*d*=0.79, PSS_T1-T3_*d*=0.6, MBI_T1-T3_*d*=0.72). Figure 2 illustrates the simple slope analysis for the moderators stress, depressive, and anxiety symptoms on their respective symptom severity.

Medium to large effect sizes were found for changes in anxiety at postassessment, with the smallest effect for participants with low emotional exhaustion (*d*=0.56; 95% CI 0.22-0.91; NNT=3.25) and the largest effect for participants with high levels of emotional exhaustion at baseline (*d*=1.19; 95% CI 0.81-1.57; NNT=1.67). Moderators of change in the outcome anxiety at postassessment were stress (beta=0.47, *P*=.036) and emotional exhaustion (beta=0.65, *P*=.002). Individuals with initially higher levels of emotional exhaustion showed significantly higher effectiveness in reducing anxiety (low: MBI_T1-T2_*d*=0.56, high: MBI_T1-T2_*d*=1.19). Individuals with low stress benefitted slightly more than individuals with moderate or high stress in reducing anxiety; however, all effects are considered large (low: PSS_T1-T2_*d*=0.88, moderate: PSS_T1-T2_*d*=0.8, high: PSS_T1-T2_*d*=0.81).

Effects in change of anxiety were moderate to large at 6-month follow-up; participants with low emotional exhaustion at baseline showed smaller effects (*d*=0.7; 95% CI 0.36-1.05; NNT=2.63) than participants with high emotional exhaustion (*d*=1.21; 95% CI 0.83-1.6; NNT=1.64). Anxiety was moderated by stress (beta=0.52, *P*=.034), emotional exhaustion (beta=0.52, *P*=.027), and anxiety (beta=0.59, *P*=.013); individuals with higher initial symptom severity stress, anxiety, and emotional exhaustion were profiting more from the intervention (low: PSS_T1-T3_*d*=0.99; HADS_T1-T3_*d*=0.76, MBI_T1-T3_*d*=0.7; high: PSS_T1-T3_*d*=1.17, HADS_T1-T3_*d*=1.16, MBI_T1-T3_*d*=1.21). With regard to differences in change from baseline to 6-month follow-up, effects in all investigated subgroups of different initial symptom severity were significant, as shown in Table 4, and in favor of the iSMI compared with the WLC.

**Table 3:** Effects on change in perceived stress, depressive symptoms, and anxiety from baseline to postassessment for different subgroups of baseline psychopathology severity (Mean−1SD, Mean, and Mean+1SD).

Moderators at postassessment			Mean–1SD		Mean		Mean+1SD	
		*P*^a^ value	*d*^b^ (95% CI)	NNT^c^	*d*^b^ (95% CI)	NNT^c^	*d*^b^ (95% CI)	NNT^c^
**Outcome: stress**								
	MOD_Stress^d^ (PSS)^e^	.01	0.65 (0.29-1)	2.82	0.98 (0.8-1.16)	1.95	0.98 (0.63-1.33)	1.95
	MOD_Depression^f^(CES-D)^g^	.11	0.82 (0.47-1.18)	2.28	0.85 (0.67-1.03)	2.21	1.05 (0.67-1.42)	1.85
	MOD_Anxiety^h^ (HADS)^i^	.61	0.79 (0.42-1.17)	2.36	0.86 (0.69-1.04)	2.19	0.86 (0.5-1.22)	2.19
	MOD_EmotExhaustion^j^ (MBI)^k^	.008	0.57 (0.22-0.91)	3.18	0.86 (0.69-1.04)	2.19	1.16 (0.78-1.54)	1.7
	MOD_Insomnia^l^ (ISI)^m^	.87	1.14 (0.77-1.51)	1.72	0.8 (0.62-0.98)	2.34	0.88 (0.53-1.22)	2.15
**Outcome: depression**								
	MOD_Stress (PSS)	<.001	0.49 (0.14-0.84)	3.68	0.68 (0.51-0.86)	2.7	0.92 (0.57-1.26)	2.07
	MOD_Depression (CES-D)	.001	0.67 (0.32-1.02)	2.75	0.7 (0.53-0.88)	2.63	1.08 (0.7-1.46)	1.81
	MOD_Anxiety (HADS)	.38	0.76 (0.39-1.14)	2.44	0.67 (0.5-0.85)	2.75	0.69 (0.33-1.04)	2.67
	MOD_EmotExhaustion (MBI)	<.001	0.37 (0.03-0.71)	4.85	0.66 (0.49-0.84)	2.78	1.01 (0.63-1.38)	1.91
	MOD_Insomnia (ISI)	.11	0.66 (0.31-1.01)	2.78	0.64 (0.47-0.82)	2.86	0.82 (0.48-1.16)	2.28
**Outcome: anxiety**								
	MOD_Stress (PSS)	.04	0.88 (0.52-1.25)	2.15	0.8 (0.62-0.98)	2.34	0.81 (0.47-1.16)	2.3
	MOD_Depression (CES-D)	.55	1.04 (0.68-1.4)	1.86	0.75 (0.57-0.93)	2.48	0.89 (0.52-1.26)	2.13
	MOD_Anxiety (HADS)	.09	0.58 (0.21-0.95)	3.14	0.93 (0.76-1.11)	2.04	0.79 (0.43-1.14)	2.36
	MOD_EmotExhaustion (MBI)	.002	0.56 (0.22-0.91)	3.25	0.77 (0.6-0.95)	2.42	1.19 (0.81-1.57)	1.67
	MOD_Insomnia (ISI)	.28	0.98 (0.62-1.34)	1.95	0.69 (0.51-0.87)	2.67	1.12 (0.77-1.47)	1.75

^a^*P* values of the standardized regression coefficient of the interaction effect between the potential moderator and group (iSMI and WLC).

^b^Cohen *d*.

^c^NNT: number needed to treat.

^d^MOD_Stress: moderator stress.

^e^PSS: Perceived Stress Scale.

^f^MOD_Depression: moderator depression.

^g^CES-D: Center for Epidemiological Depression Scale.

^h^MOD_Anxiety: moderator anxiety.

^i^HADS: Hospital Anxiety and Depression Scale.

^j^MOD_EmotExhaustion: moderator emotional exhaustion.

^k^MBI: Maslach Burnout Inventory.

^l^MOD_Insomnia: moderator insomnia severity.

^m^ISI: Insomnia Severity Index.

### Response Rates and Symptom-Free Status in Clinical Subgroups

Furthermore, subgroups were investigated in which individuals had values indicating clinical depression levels at baseline (CES-D≥16, 82.9%, 656/791, mean 26.36, SD 7.19). At postassessment, the iSMI group (n=318) had a mean CES-D value of mean 17.76 (SD 8.69) and a CES-D change score of M_T1-T2_=9.05 (SD_T1-T2_ 8.79) compared with the WLC (n=338, mean 23.1, SD 8.83, M_T1-T2_=2.85, SD_T1-T2_ 7.43). This resulted in a moderate to large effect of Cohen *d*=0.76 (95% CI 0.6-0.92) and an NNT of 2.44. On the basis of the CES-D, a score reduction of 50% from baseline to postassessment was achieved significantly more often in participants of the iSMI (CES-D: 29.2%, 93/318) as compared with the WLC (CES-D: 8.0%, 27/338; χ^2^_1_=49.5; *P*<.001; NNT=4.7), and significantly more individuals in the iSMI compared with the WLC met criteria for SF status (CES-D<16) at postassessment (iSMI: CES-D: 39.6%, 126/318; WLC: 18.6%, 63/338; χ^2^_1_=35.2; *P*<.001; NNT=4.8).

**Table 4:** Effects on change in perceived stress, depressive symptoms, and anxiety from baseline to 6-month follow-up for different subgroups of baseline psychopathology severity (mean-1SD, mean, and mean+1SD).

Moderators at 6-month follow-up			Mean-1SD		Mean		Mean+1SD	
		*P*^a^ value	*d*^b^ (95% CI)	NNT^c^	*d*^b^ (95% CI)	NNT^c^	*d*^b^ (95% CI)	NNT^c^
**Outcome: stress**								
	MOD_Stress^d^ (PSS)^e^	.049	0.66 (0.3-1.02)	2.78	0.91 (0.73-1.09)	2.08	1.27 (0.9-1.63)	1.59
	MOD_Depression^f^(CES-D)^g^	.12	0.7 (0.35-1.05)	2.63	0.92 (0.74-1.1)	2.07	1.05 (0.67-1.42)	1.85
	MOD_Anxiety^h^ (HADS)^i^	.55	0.91 (0.53-1.29)	2.08	0.85 (0.67-1.03)	2.21	0.93 (0.57-1.29)	2.04
	MOD_EmotExhaustion^j^ (MBI)^k^	.05	0.8 (0.45-1.15)	2.34	0.78 (0.61-0.96)	2.39	1.35 (0.96-1.75)	1.52
	MOD_Insomnia^l^ (ISI)^m^	.53	1.02 (0.65-1.38)	1.89	0.79 (0.61-0.97)	2.36	1.05 (0.7-1.4)	1.85
**Outcome: depression**								
	MOD_Stress (PSS)	.001	0.6 (0.24-0.95)	3.05	0.77 (0.59-0.94)	2.42	1.2 (0.84-1.56)	1.66
	MOD_Depression (CES-D)	.025	0.79 (0.44-1.15)	2.36	0.94 (0.76-1.12)	2.02	1.02 (0.65-1.39)	1.89
	MOD_Anxiety (HADS)	.33	0.9 (0.52-1.28)	2.1	0.77 (0.6-0.95)	2.42	0.91 (0.55-1.27)	2.08
	MOD_EmotExhaustion (MBI)	.002	0.72 (0.37-1.07)	2.56	0.73 (0.56-0.91)	2.54	1.19 (0.81-1.58)	1.67
	MOD_Insomnia (ISI)	.11	0.78 (0.43-1.14)	2.39	0.77 (0.59-0.95)	2.42	0.86 (0.52-1.21)	2.19
**Outcome: anxiety**								
	MOD_Stress (PSS)	.034	0.99 (0.63-1.36)	1.94	0.84 (0.66-1.02)	2.23	1.17 (0.81-1.53)	1.69
	MOD_Depression (CES-D)	.36	1.11 (0.75-1.48)	1.76	0.87 (0.69-1.05)	2.16	1.06 (0.69-1.44)	1.83
	MOD_Anxiety (HADS)	.013	0.76 (0.38-1.13)	2.44	1.01 (0.84-1.19)	1.91	1.16 (0.78-1.53)	1.7
	MOD_EmotExhaustion (MBI)	.027	0.7 (0.36-1.05)	2.63	0.89 (0.71-1.07)	2.13	1.21 (0.83-1.6)	1.64
	MOD_Insomnia (ISI)	.42	1.04 (0.68-1.4)	1.86	0.82 (0.64-1)	2.28	1.06 (0.71-1.41)	1.83

^a^*P* values of the standardized regression coefficient of the interaction effect between the potential moderator and group (iSMI and WLC).

^b^Cohen *d*.

^c^NNT: number needed to treat.

^d^MOD_Stress: moderator stress.

^e^PSS: Perceived Stress Scale.

^f^MOD_Depression: moderator depression.

^g^CES-D: Center for Epidemiological Depression Scale.

^h^MOD_Anxiety: moderator anxiety.

^i^HADS: Hospital Anxiety and Depression Scale.

^j^MOD_EmotExhaustion: moderator emotional exhaustion.

^k^MBI: Maslach Burnout Inventory.

^l^MOD_Insomnia: moderator insomnia severity.

^m^ISI: Insomnia Severity Index.

Individuals with clinically relevant levels of anxiety at baseline (HADS≥8, 85.3%, 675/791, mean 11.94, SD 2.82) showed a large effect of Cohen *d*=0.87 (95% CI 0.72-1.03) in favor of the iSMI at postassessment. Individuals in the iSMI (n=339) had a mean HADS value of mean 8.15 (SD 3.65) and HADS change score M_T1-T2_=3.81 (SD_T1-T2_ 3.3) compared with the WLC (n=336, mean 10.8, SD 3.38, M_T1-T2_=1.13, SD_T1-T2_ 2.81). Treatment response, that is, symptom reduction of 50% in the HADS, was assessed significantly more often in the iSMI (27.7%, 94/339) compared with the WLC (4.8%, 16/336; χ^2^_1_=65.2; *P*<.001; NNT=4.4). SF status (HADS<8) was assessed significantly more often in the iSMI (39.8%, 135/339) compared with the WLC (15.5%, 52/336; χ^2^_1_=49.9; *P*<.001; NNT=4).

## Discussion

### Principal Findings

This study aimed to explore whether iSMIs are effective in severely burdened employees and tested whether baseline indicators of impairment moderated treatment outcome. Highly burdened participants who showed high levels of stress, depression, anxiety, emotional exhaustion, or insomnia severity profited substantially from the intervention on all outcome measures with moderate to large intergroup effect sizes compared with the control condition, both at postassessment and at 6-month follow-up. Moreover, higher impairment (ie, depression, anxiety, perceived stress, and emotional exhaustion) was associated with greater symptom improvement over time. These findings are in line with (1) studies showing that internet-based self-help interventions can be effective in clinical populations [38-40], (2) studies showing that internet-based occupational health interventions specifically designed to target depression can be effective [41,42], and (3) moderator analyses showing that higher symptom severity is not associated with worse treatment outcome in low-threshold self-help interventions [19,43]. This study extends these findings by showing that highly burdened participants with high levels of stress, depression, anxiety, insomnia severity, or emotional exhaustion can also substantially profit from an intervention that is labelled and specifically designed to reduce negative consequences of occupational stress. Reasons may include that the iSMI contains techniques based on cognitive behavioral therapy (ie, problem-solving and emotion regulation), which have shown to be effective in treating depression and anxiety [21,44].

Many of the participants were first-time help-seekers, indicated by 86.5% having no prior experience with health-related training and less than half (39.3%) having prior experience with face-to-face therapy, although the majority of participants had already reached clinically relevant levels of depression (82.9%) and anxiety (85.3%) before study uptake. This indicates that stress management may be a valid entry point to reach highly burdened individuals who otherwise may not seek treatment. Stress is less stigmatized than depression and anxiety, and online treatment provides the necessary anonymity for uncertain individuals to initially seek help.

Individuals of subgroups with high symptom severity at baseline (CES-D≥33; HADS≥15; PSS≥30; ISI≥20; MBI≥5.6) experienced significant reductions with large intergroup effect sizes, which were also sustained over time (6-month follow-up: depression, *d* [0.86-1.19]; anxiety *d* [1.06-1.21], stress *d* [0.93-1.27]). Although significantly more individuals in the iSMI intervention group were assessed to have reached a RR of at least 50% symptom reduction (CES-D: 29.2%, 93/318; HADS: 27.7%, 94/339) and even SF status (CES-D: 39.6%, 126/318; HADS: 39.8%, 135/339), many highly burdened individuals, however, did not achieve either. This indicates that iSMIs cannot fully substitute more intensive psychological treatment for all individuals with high levels of anxiety or depression. Such findings highlight the importance of monitoring individual progress throughout treatment to refer individuals to further, more intensive or simply different treatment modalities, based on different theoretical constructs or treatment formats after intervention completion. Another possibility may be to tailor the treatment to the individual symptom profile if the participant does not respond sufficiently to the standardized treatment. However, future studies are needed to explore if such approaches indeed lead to better treatment outcome, as effects were already large in terms of effect sizes, and it is also possible that there is a limit to what can be achieved with psychological interventions.

### Limitations

The study also has some limitations. First, as common for randomized trials, there was an elaborate screening process at study entry. This may have caused individuals with lower self-efficacy and less motivation to dropout before study uptake, and those most likely to profit to continue. Second, considering the description of the study population, the variance was rather low, reducing the heterogeneity of variance to explore differences within subgroups of the study sample. Third, subgroups were created based on the mean and 1 SD above or below the mean, which is typical for discerning simple slopes. Ranges are therefore based on this study population and may vary for other populations. Fourth, overall participants had elevated symptoms of impairment because of the study inclusion criterion heightened stress (PSS≥22). Therefore, there was no exploration of individuals with very low symptom severity. Fifth, all exploration of subgroups was based on the same iSMI GET.ON Stress. Therefore, the findings may not be generalizable directly to other interventions, especially as the latest meta-analysis on iSMIs indicated high heterogeneity between studies and interventions [5]. Finally, the main finding of this study that individuals with severe impairment profit greatly from the iSMI cannot be generalized to individuals who show indications of suicidality, as the studies excluded individuals with high suicide risk at baseline. Unfortunately, this remains an unresolved issue many internet interventions face, how to adequately deal with at-risk individuals who show active interest in participation but whose monitoring throughout a low-threshold intervention, often, cannot be guaranteed.

Strengths of this study include the strong methodology of RCTs, and the pooling of individual participant data from different studies investigating the same intervention, hereby overcoming the issue of studies generally being underpowered to explore subgroups [16].

### Conclusions

This study contains important implications for research and practice. First, populations who experience high stress, are clinically depressed, or have high anxiety can alleviate their disease burden and reduce symptom severity by participating in iSMIs. Second, occupational stress and stress management are effective entry points for initial contact with mental health interventions, and therefore, severely burdened individuals should be targeted for participation. Third, future studies should explore whether tailoring of iSMIs to clinical profiles leads to superior outcomes in severely affected individuals compared with standardized approaches. Finally, as employers benefit from healthy employees, they should consider offering iSMIs in routine occupational settings and encourage participation. Severely burdened employees, especially those with high levels of depression and anxiety, should not be excluded but rather motivated to participate in iSMIs.

## Abbreviations

CES-D: Center for Epidemiological Depression Scale
HADS: Hospital Anxiety and Depression Scale
ISI: Insomnia Severity Index
iSMI: internet-based and mobile-based stress management interventions
MBI: Maslach Burnout Inventory
MCAR: missing completely at random
MI: multiple imputations
MMAs: multiple moderation analyses
NNT: number needed to treat
PSS: Perceived Stress Scale
RCT: randomized controlled trial
RR: response rate
SF: symptom-free
SMD: standardized mean difference
WLC: waitlist control group
